# Ultrasonographic kidney length-to-abdominal aortic diameter for the diagnosis of feline chronic kidney disease: A preliminary study

**DOI:** 10.14202/vetworld.2023.1114-1121

**Published:** 2023-05-27

**Authors:** Kotchapol Jaturanratsamee, Nan Choisunirachon, Kumpanart Soontornvipart, Damri Darawiroj, Naparee Srisowanna, Chutimon Thanaboonnipat

**Affiliations:** 1 Department of Surgery, Faculty of Veterinary Science, Chulalongkorn University, Bangkok, Thailand; 2 Department of Anatomy, Faculty of Veterinary Science, Chulalongkorn University, Bangkok, Thailand; 3 Department of Anatomy, Faculty of Veterinary Medicine, Kasetsart University, Bangkok, Thailand

**Keywords:** cats, chronic kidney disease, kidney length-to-abdominal aortic diameter, renal length, ultrasonography

## Abstract

**Background and Aim:**

Chronic kidney disease (CKD) is one of the most important diseases in cats. This study aimed to compare the ultrasonographic kidney length-to-abdominal aortic diameter (K/AO) ratio between healthy and CKD cats and investigate the correlation between K/AO and blood results.

**Materials and Methods:**

Fifteen healthy cats and 15 CKD cats were included in this clinically prospective study. All cats were evaluated for radiographic and ultrasonographic K, radiographic K-to-second lumbar length ratio (K/L2), and K/AO, indirect systolic blood pressure and plasma creatinine (Cr), blood urea nitrogen (BUN), and symmetric dimethyl arginine (SDMA).

**Results:**

The radiographic and ultrasonographic kidney lengths of CKD were significantly shorter than those of healthy cats (p < 0.05 and p < 0.05, respectively). The average K/L2 and K/AO were significantly lower in CKD than in healthy cats (p < 0.01 and p < 0.001, respectively). The K/AO had a strong negative correlation with plasma Cr (r = −0.7682, p < 0.0001), BUN (r = −0.6175, p < 0.001), and SDMA (r = −0.589, p < 0.001). However, K/L2 had a moderate negative correlation with plasma Cr (r = −0.5866, p < 0.001), BUN (r = −0.4884, p < 0.01), and SDMA (r = −0.5404, p < 0.01). The optimal cutoff value of K/AO (<10.71) had higher sensitivity and specificity than K/L2 for identifying feline CKD.

**Conclusion:**

Kidney length-to-abdominal aortic diameter could be a better and more promising parameter than the K/L2 ratio for evaluating kidney size in cats with CKD.

## Introduction

Chronic kidney disease (CKD) is defined as the presence of structural or functional abnormalities in either one or both kidneys for a period longer than 3 months. Chronic kidney disease can manifest itself in a variety of ways, ranging from a slight anatomical lesion in one kidney to a complete loss of nephrons in both kidneys. Chronic kidney disease patients may present with many clinical signs, diagnostic findings, and treatment issues [1], and CKD in cats is one of the most common metabolic diseases in clinical practice [2]. Although CKD has been reported in cats of all ages, the prevalence of this condition has been increased in senile cats [3]. Chronic kidney disease can be diagnosed with history taking, physical examination, and biochemical tests. In clinical practice, blood creatinine (Cr) concentration is widely used as a surrogate marker for glomerular filtration rate evaluation. However, this technique has several limitations, and its value can be affected by other non-renal factors [4].

Ultrasonography is one of the most common diagnostic imaging methods used in clinical practice [5]. More specifically, this useful diagnostic tool can evaluate and detect renal parenchyma alteration [2] occurring with disease progression. The previous report has shown that the kidney of CKD cats is characterized by significantly decreased size and contour changes [1]. Ultrasonographic findings that are normally found in CKD cats include decreased renal size, irregular contour, increased parenchymal echogenicity, loss of corticomedullary demarcation, mineralization, and poor identification of internal structures [6, 7]. The normal kidney length (K) in cats is reported to be in the range of 2.80–4.40 cm [7]. However, this range is not only wide but may also be unsuitable for generalization to all cats due to their different body sizes. A reference range for estimating the renal size of dogs has been reported by calculating the K-to-abdominal aortic diameter (K/AO) ratio [8], whose normal range is 5.50–9.10 [8]. However, due to the wide range and non-specificity of this value due to various canine body sizes, many studies have recently reported a canine breed-specific K/AO for Whippet [9] and Miniature Schnauzer [10]. At present, only one study has reported the K/AO for normal cats ranging between 9.80 and 12.60 [11]. However, the K/AO in CKD cats is still lacking, and the cutoff values for ultrasonographic estimation of renal size for the diagnosis of CKD cats have yet to be reported.

Therefore, this study aimed to compare the K/AO between healthy and CKD cats using ultrasonography and investigate the correlation between K/AO and blood biochemical results, including Cr and symmetric dimethyl arginine (SDMA) concentrations.

## Materials and Methods

### Ethical approval and informed consent

This study was designed as a prospective study and was approved by the institutional guidelines and conformed to the Chulalongkorn University Animal Care and Use Committee (CU-ACUC), approval number: 2131022. All experiments were conducted in accordance with institutional guidelines and regulations, and the study complied with the Animal Research: Reporting of *in vivo* Experiments guidelines [12]. Informed consent (either verbal or written) was obtained from the owner or legal custodian of all animals described in this work for the procedures undertaken.

### Study period and location

The study included medical information on client-owned cats that were presented to the Diagnostic Imaging Unit, The Small Animal Hospital, Faculty of Veterinary Science, Chulalongkorn University from August 2021 to March 2022.

### Experimental design

In this prospective study, cats were divided into two groups, namely, healthy (n = 15) and CKD (n = 15) groups, depending on their medical history, physical examination, and the International Renal Interest Society (IRIS) staging system. All cats included in the CKD group were defined based on a history of either structural or functional abnormalities of the kidneys for a period more than 3 months (stable CKD patients) and were categorized into IRIS stages 2–4 according to the IRIS staging system [13] in which plasma Cr concentrations are equal to or higher than 1.6 mg/dL. All cats with urinary obstruction and congenital kidney diseases, such as polycystic kidney disease, hydronephrosis, pyelonephritis, renal tumor, and big kidney-small kidney syndrome, were excluded from this study.

### Experimental protocols

All cats (both healthy and CKD) included in this study were mature (more than 1 year old) with normal hydration status. Cats with congenital renal abnormality, renal tumors on ultrasonographic examination, hypertensive (systolic blood pressure more than 160 mmHg), or pregnant cats were excluded from the study. Enrolled cats were examined to confirm their physical condition through general appearance, mentation, hydration status, temperature, heart rate and rhythm, respiratory rate, mucous membrane color, capillary refill time, lung sounds, and abdominal palpation, and the examination results were then recorded. All cats had blood collected (3 mL) through the cephalic vein or femoral vein in tripotassium ethylene diamine tetraacetic acid (K3) and lithiumheparin tubes (South tech lab, Bangkok, Thailand). Common hematology and basic blood biochemistry, including alkaline phosphatase, gamma-glutamyl transferase, blood urea nitrogen (BUN), Cr, and SDMA, were performed in all cats to confirm their health status and CKD staging procedures. The cat was excluded from this study if there were any other diseases. Systolic blood pressure was measured indirectly using a Doppler device (BV-520, Shenzhen Bestman Instrument, Shenzhen, China). Cats had their hair over the palmar aspect of the carpus removed, and the Doppler probe was then placed perpendicularly over the digital artery. Cuff measurements had a width of 30%–40% of that of the leg circumference [14]. All cats were measured 3 times and the results were averaged. A value of more than 160 mmHg was the criterion for normal blood pressure [13].

### Experimental procedures

#### Radiographic procedures and kidney size evaluation

Right lateral and ventrodorsal (VD) abdominal radiographs of all cats were obtained without sedation using a digital radiography system (ETL®, GE Healthcare, Chicago, USA). All cats were manually restrained and positioned during radiography to obtain a suitable radiograph during the expiratory phase. All radiographs were saved as Digital Imaging and Communications in Medicine (DICOM) format was evaluated using DICOM viewer software (OSIRIX®, Geneva, Switzerland) with a digital caliper. In brief, the K on a VD radiograph was measured from the cranial pole to the caudal pole. It was then compared with the second lumbar vertebral length (L2) measured at the midline vertebral body in accordance with the Barrett and Kneller technique [15]. Consequently, the K-to-second lumbar length ratio (K/L2) was calculated.

#### Ultrasonographic procedures and kidney size evaluation

**Figure-1:**
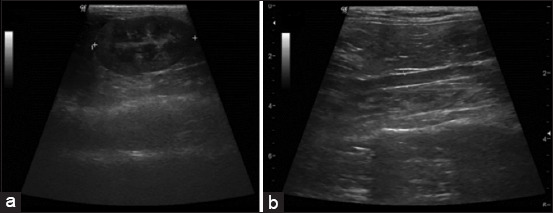
(a) The measurement of kidney length and (b) abdominal aorta diameter on sagittal plane.

All cats were fasted for 8 h before the procedures. All cats were mechanically restrained in the VD position and their hair was clipped. Before the examination, an acoustic coupling gel was applied to the skin. A general survey ultrasound was primarily performed for general observation of the whole abdomen using a 9 MHz linear transducer with real-time B-mode (Logiq P6 ultrasound machine, GE Healthcare, Korea). Subsequently, the K was measured from the cranial pole to the caudal pole (K; Figure-1a), and the aortic luminal diameter (AO; Figure-1b) was measured at the caudal area to the origin of the left renal artery in accordance with the technique presented by Mareschal *et al*. [8]. The K/AO was then calculated. All parameters were measured in triplicate and the results were averaged.

### Statistical analysis

GraphPad Prism 9 software (GraphPad Software, CA, USA) was used to perform all statistical analyses. All data were expressed as mean ± SD. The D’Agostino-Pearson omnibus and Shapiro-Wilk normality tests were used to evaluate the normality of the dataset. The Mann–Whitney U test was used to compare renal function parameters between healthy and CKD cats. An unpaired t-test was used to compare indirect systolic blood pressure and kidney size parameters between healthy and CKD cats and male and female cats. A paired t-test was used to compare K between radiographs and ultrasonographic images at the left and right sides. One-way analysis of variance was used to compare the kidney size parameters among healthy cats, cats with an early and late IRIS stage. p < 0.05 was considered statistically significant.

## Results

### Clinical and demographic characteristics

Fifteen healthy cats and 15 CKD cats were enrolled in this study. The healthy cat group consisted of domestic shorthair (n = 8 [53.33%]), British short hair (n = 5 [33.33%]), and Scottish fold (n = 2 [13.33%]), while the CKD group consisted of domestic shorthair (n = 13 [86.67%]), British short hair (n = 1 [6.67%]), and Scottish fold (n = 1 [6.67%]). The clinical demographic data such as sex, gonadal status, age, body weight (BW), and renal function parameters are reported in Table-1. There were no significant differences between the sexes in healthy and CKD cats (p > 0.9999). Chronic kidney disease cats were significantly older than healthy cats (p = 0.0028). The average BW of healthy and CKD cats was 4.33 ± 0.92 kg (range 2.90–5.90) and 4.16 ± 1.04 kg (range 3.00–7.05), respectively. The CKD cats were divided into the early IRIS stage (IRIS stage 2) (n = 11 [73.00%]) and the late IRIS stage (IRIS stage 3–4) (n = 4 [27.00%]). The average indirect systolic blood pressure between healthy and CKD cats was not significantly different (p = 0.3814). Plasma concentrations of Cr, BUN, and SDMA of the CKD cats were significantly higher than those of the healthy cats (p < 0.0001, p = 0.0022, and p = 0.0053, respectively).

**Table-1:** Clinical demographic information and renal function parameters include plasma concentrations of Cr, BUN, SDMA, and indirect systolic blood pressure of all included cats.

Parameters	Healthy cats (n = 15)	CKD cats (n = 15)
Sex		
Female	9	8
Intact	5	1
Neutered	4	7
Male	6	7
Intact	2	2
Neutered	4	5
Age (years)	4.60 ± 2.97 (1.00–10.00)	9.53 ± 5.01^++^ (1.00–19.00)
BW (kg)	4.33 ± 0.92 (2.90–5.90)	4.16 ± 1.05 (3.00–7.05)
Plasma Cr (mg/dL)	1.33 ± 0.33	2.81 ± 1.60****
BUN (mg/dL)	23.51 ± 4.01	39.05 ± 24.87**
SDMA (µg/dL)	14.72 ± 2.81	22.87 ± 10.04**
Indirect systolic blood pressure (mmHg)	144.20 ± 29.73	131.60 ± 28.38

Statistically significant difference between groups was made using Mann–Whitney U test,

** p < 0.01;

**** p < 0.0001 Statistically significant difference between groups was made using unpaired t-test,

++ p *<* 0.01 Data are presented as mean ± SD. Cr=Creatinine, BUN=Blood urea nitrogen, SDMA=Symmetric dimethyl arginine, BW=Body weight

### Kidney size parameters were observed through radiography and ultrasonography

**Figure-2:**
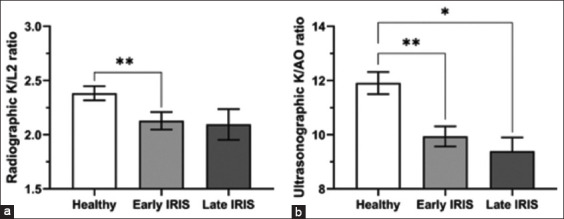
(a) Radiographic average kidney length-to-second lumbar length ratio and (b) ultrasonographic average kidney length-to-abdominal aortic diameter ratio among healthy cats, cats with early and late International Renal Interest Society stages.

In this study, the radiographic left kidney (LK) length could be measured in all cats, whereas the radiographic right kidney (RK) length could be measured in 9/15 (60%) healthy cats and 14/15 (93%) CKD cats. In each group, the LK and RK lengths were not significantly different (p = 0.1720). The average K in healthy cats was significantly higher than that in CKD cats (p = 0.0256; Table-1). No significant difference of L2 length was detected between the two groups (p = 0.1336). With respect to the K/L2 ratios, both LK/L2 and average K/L2 in healthy cats were significantly higher than those in CKD cats (p = 0.0022 and p = 0.0089, respectively; Table-2). In contrast, the RK/L2 ratio was not significantly different between the two groups (p = 0.1752). Furthermore, the average K/L2 of healthy cats was significantly higher than that of cats with early and late IRIS stages (p = 0.0220), and the K/L2 ratio was not significantly different between cats with early and late IRIS stages (p = 0.8258; Figure-2a).

**Table-2:** Kidney size parameters including radiographic K, L2, ultrasonographic K, AO then calculated K/L2 in radiographic parameters, and K/AO ratio in ultrasonographic parameters of healthy and CKD cats.

Parameters	Radiography			Ultrasonography		
	
	Kidney length (cm)	L2 (cm)	K/L2	Kidney length (cm)	AO (cm)	K/AO
Healthy cats	4.09 ± 0.50	1.74 ± 0.10	2.38 ± 0.25	3.72 ± 0.46	0.31 ± 0.03	11.91 ± 1.59
	(3.82–4.37)	(1.68–1.79)	(2.24–2.52)	(3.47–3.98)	(0.30–0.33)	(11.03–12.79)
CKD cats	3.79 ± 0.42*	1.80 ± 0.13	2.12 ± 0.27**	3.33 ± 0.41*	0.34 ± 0.05	9.80 ± 1.17***
	(3.57–4.03)	(1.73–1.87)	(1.97–2.26)	(3.10–3.56)	(0.32–0.37)	(9.15–10.44)

Statistically significant difference between groups was made using unpaired t-test,

* p < 0.05;

** p < 0.01;

*** p < 0.001. Data are presented as mean ± SD. K=Kidney length, L2=Second lumbar vertebral length, AO=Abdominal aortic diameter, K/L2=Kidney length-to-second lumbar length ratio, K/AO=Kidney length-to-aortic diameter, CKD=Chronic kidney disease, SD=Standard deviation

Ultrasonographic K could be measured in all kidneys of both groups. The ultrasonographic appearances of the kidneys in the healthy group had normal shape, contour, echogenicity, and echotexture. In contrast, CKD cats had irregular contours, increased echogenicity of the renal cortex and/or medulla, and decreased corticomedullary demarcation. All ultrasonographic parameters are presented in Table-2. There was no significant difference in the K of the left and right sides in each group (p = 0.2734). Chronic kidney disease cats had significantly shorter LK and average K lengths than healthy cats (p = 0.0041 and p = 0.0189, respectively). The RK length was not significantly different between healthy and CKD cats (p = 0.1002). Moreover, the AO diameter of both groups was not significantly different (p = 0.0577), and the K/AO ratio between the left and RKs in each group was not significantly different (p = 0.2847). Both LK/AO and RK/AO ratios of healthy cats were significantly higher than those of CKD cats (p < 0.0001 and p = 0.0036, respectively). Finally, the K/AO ratio of the healthy group was significantly higher than that of cats with early and late IRIS stages (p = 0.0044 and p = 0.0103, respectively), and the average K/AO of the early IRIS stage group was not significantly different from that in the late IRIS stage group (p = 0.7865; Figure-2b).

### Correlations between kidney size parameters and functional renal parameters

**Figure-3:**
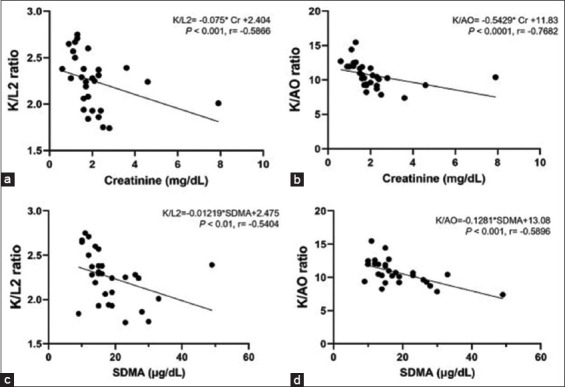
The linear regression graph of either kidney length-to-second lumbar length ratio (K/L2) or kidney length-to-abdominal aortic diameter (K/AO) and plasma concentrations of Creatinine (Cr) and Symmetric dimethyl arginine (SDMA). (a) Average K/L2 and plasma Cr concentration. (b) Average K/AO and plasma Cr concentration. (c) Average K/L2 ratio and SDMA. (d) Average K/AO and SDMA.

The correlation between kidney size parameters and functional renal parameters, including the concentrations of plasma Cr, BUN, and SDMA of all cats, is shown in Table-3. All kidney size parameters had significantly negative correlations with functional renal parameters. The average K/L2 ratio had a significant negative correlation with plasma Cr (r = −0.5866, p = 0.0007), BUN (r = −0.4884, p = 0.0062), and SDMA (r = −0.5404, p = 0.0021). The average K/AO ratio had a significant negative correlation with the concentrations of plasma Cr (r = −0.7682, p < 0.0001), BUN (r = −0.6175, p = 0.0003), and SDMA (r = −0.589, p = 0.0006; Figure-3). The average K from both radiographic and ultrasonographic images was also significantly negatively correlated with plasma Cr (r = −0.4970, p = 0.0052 and r = −0.4890, p = 0.0061, respectively), BUN (r = −0.4283, p = 0.0182 and r = −0.4231, p = 0.0198, respectively), and SDMA (r = −0.4483, p = 0.0130 and r = −0.4061, p = 0.0260, respectively).

**Table-3:** Correlations between kidney size parameters and plasma concentrations of Cr, BUN and SDMA of the healthy and CKD cats.

Parameters	Radiography		Ultrasonography	
	
	Average kidney length r	Average K/L2 ratio r	Average kidney length R	Average K/AO ratio r
Plasma Cr	−0.4970**	−0.5866***	−0.4890**	−0.7682****
BUN	−0.4283*	−0.4884**	−0.4231*	−0.6175***
SDMA	−0.4483*	−0.5404**	−0.4061*	−0.5896***

Correlations between parameters were made using Spearman correlation,

* p < 0.05;

** p < 0.01;

*** p < 0.001;

**** p < 0.0001. Cr=Creatinine, BUN=Blood urea nitrogen, SDMA=Symmetric dimethyl arginine, K/AO=Kidney length-to-abdominal aortic diameter, K/L2=Kidney length-to-second lumbar length ratio

### Receiver operating characteristic (ROC) curve and the area under the ROC curve of kidney size parameters

The optimal cutoff values (Youden index) and associated sensitivity, specificity, and likelihood ratio of each imaging parameter are presented in Table-4. The K/L2 ratio cutoff value was 2.27 with sensitivity and specificity for identifying 67% and 73% CKD cats, respectively (K/L2 ratio ≤2.27). The maximum specificity cutoff value for the K/L2 ratio was 2.04, with 47% sensitivity and 93% specificity, while the optimal cutoff value for the K/AO ratio was 10.71, with sensitivity and specificity for identifying CKD cats of 80% and 80%, respectively, (K/AO ratio ≤10.71). Finally, the maximum specificity cutoff value for the K/AO ratio was 9.99 with 60% sensitivity and 93% specificity.

**Table-4:** Results of ROC curve analysis of all kidney size parameters.

Imaging parameters	ROC curve analysis		Cutoff value		Percentage sensitivity (CI 95%)	Percentage specificity (CI 95%)
	
	AUC (95% CI)	p-value	Type	cutoff		
Radiography						
Average kidney length (cm)	0.68 (0.487–0.873)	0.093	Optimal sensitivity	3.85	60 (32–84)	73 (45–92)
			Maximum specificity	3.48	20 (4–48)	93 (68–100)
K/L2 ratio	0.77 (0.604–0.938)	<0.05	Optimal sensitivity	2.27	67 (33–88)	73 (45–92)
			Maximum specificity	2.04	47 (21–73)	93 (68–100)
Ultrasonography						
Average kidney length (cm)	0.72 (0.532–0.908)	<0.05	Optimal sensitivity	3.53	67 (38–88)	73 (45–92)
			Maximum specificity	3.06	20 (4–48)	93 (68–100)
K/AO ratio	0.87 (0.746–0.997)	<0.001	Optimal sensitivity	10.71	80 (52–96)	80 (52–96)
			Maximum specificity	9.99	60 (32–84)	93 (68–100)

ROC=Receiver operating characteristic, CI=Confidence interval, AUC=Area under the curve, K/AO=Kidney length-to-abdominal aortic diameter, K/L2=Kidney length-to-second lumbar length ratio. Receiver operator characteristic analysis was used to identify optimal cut off values. Optimal sensitivity cut off value was determined with the Youden index and likelihood ratio. Maximum specificity cut off value with near 100% specificity

## Discussion

The aim of this study was to compare the K/AO value between healthy and CKD cats using ultrasonography and investigate the correlations between K/AO and blood biochemical results, including Cr and SDMA concentrations. In veterinary medicine, kidney size parameters from radiography and ultrasonography, including K/L2 and K/AO, have only been reported in healthy cats [11]. Therefore, this study is the first to report on the K/L2 and K/AO values in CKD cats.

In this study, the age of CKD cats was higher than that of healthy cats, which is consistent with previous reports [3, 16]. Although feline CKD has been reported in all ages [3], a higher prevalence has been found in senile cats [17], indicating that aging may contribute to the onset of CKD in affected cats [16].

Indirect systolic blood pressure was not significantly different between healthy and CKD cats in this study. Our results contradict those of previous studies in dogs [18] and cats [19], in which CKD animals had higher blood pressure than healthy ones. However, some CKD cats with hypertensive conditions in our study were treated with medication. Therefore, it might be difficult to detect an obvious difference in that case. A previous study reported a positive correlation between blood pressure and kidney volume in humans [20].

Regarding kidney radiography, K of LK could be measured in all cats in both groups. In contrast, RK could only be measured in 60% of the healthy and 93% of the CKD groups. Superimposition of kidneys with abdominal organs, especially the stomach and small intestine, affected the visualization, and precise measurement of RK length in VD abdominal radiographs [21]. Interestingly, a higher proportion of RK could be detected in CKD cats than in healthy cats. This might be caused by CKD cats having issues such as anorexia and decreased appetite [6]. Accordingly, the gastrointestinal tract of CKD cats contains a small amount of food, decreasing the superimposition effect of the gastrointestinal tract content on the kidneys. In addition, abdominal fat in CKD cats is significantly less than that in healthy cats, possibly inducing a reduced contrast of abdominal details. The radiographic K of the CKD group was significantly shorter than that of the healthy group for LK and average K. These results correspond to the results previously shown in studies on humans [22] and cats [15]. Chronic kidney disease cats are normally affected by small kidneys and contractions depending on disease progression [16]. The radiographic RK length could not be observed in all cats, possibly indicating that the LK length could be a better radiographic parameter for evaluating kidney size than the RK length. L2 length was not significantly different between the healthy and CKD groups, which could be attributed to the number of the same gender cats. It has been reported that the vertebral length could be affected by several factors, including sex [23], gonadal status, and prepubertal gonadectomy [24]. Similar to K, CKD cats had a significantly lower average K/L2 ratio than healthy cats. These results are consistent with previous reports on humans [25] and dogs [26]. Our results demonstrated that K/L2 ratios had a higher significant difference than K. A previous study reported that K was not significantly different between cats with and without azotemia [27]. Another study reported that feline K was not significantly different between the CKD and control groups [4]. Our findings showed that the K/L2 ratio was significantly different between the healthy and early CKD groups. However, there was no significant difference between the healthy and late IRIS groups or between the early IRIS and late IRIS groups. However, the sample size of cats in the late IRIS group was small, which could be a limitation of this study. Nonetheless, the 95% confidence interval of the K/L2 ratio in the healthy group was 2.24–2.52, which is outside the reference range shown in a previous study Yan *et al*. [4] reporting that the K/L2 ratio in healthy cats was 2.40–3.00. However, our results were close to the reference range reported by Shiroma *et al*. [28], which reported that the K/L2 ratios of neutered cats and intact cats were 1.9–2.6 and 2.1–3.2, respectively. However, the difference in the K/L2 range may be caused by the different proportions of gonadectomized cats in each study. Most cats in our study were neutered. Therefore, our K/L2 range was consistent with the previous findings of Shiroma *et al*. [28].

In this study, the average K of both groups on ultrasonography was less than that obtained by radiography, consistent with a previous study performed on humans [29]. Radiographic images are normally affected by the magnification and distortion of image details [30]. It has been reported that the fetal size diameter observed on radiography in both dogs and cats was larger than that measured by ultrasonography [31]. Furthermore, the ultrasonographic K of the CKD group was significantly shorter than that of the healthy group for LK and average K, which is consistent with the findings of a previous study performed in cats [4]. The mean RK length in CKD cats was lower than that in healthy cats, but there was no significant difference that might have been caused by the small number of samples in both groups. In addition, it has been reported that the size of the lesion in RK and LK did not spread equally in each CKD cat [32]. The abdominal AO diameter was not significantly different between the healthy and CKD groups. A previous study found that BW positively correlated with AO diameter but did not influence the K/AO ratio. The average K/AO ratio of CKD cats was significantly lower than that of healthy cats [11]. Moreover, the K/AO ratio was significantly different between healthy cats and cats at early IRIS stages and between healthy cats and cats at late IRIS stages. However, no significant difference was detected between the early IRIS and late IRIS groups. In addition, age and BW were not found to influence the K/AO ratio. The average K/AO ratio in the healthy group was 11.91 ± 1.59, a range that is close to that reported by Tanvetthayanont *et al*. [11], with average K/AO values of 11.69 ± 1.49 and 9.80 ± 1.17 in healthy and CSK cats, respectively.

For the correlation between kidney size parameters and plasma concentrations of Cr, BUN, and SDMA, all kidney size parameters had a negative correlation with all functional renal parameters. These results are consistent with the previous studies performed on humans [33] and cats [4]. Decreased renal size in patients with CKD is a common finding in the advanced stages of CKD [34], and small kidneys have induced functional renal deterioration and impairment [16]. The K/AO ratio had a moderate to strong negative correlation with plasma Cr, BUN, and SDMA concentrations. The K on both radiographs and ultrasonographic images had a weak negative correlation with the plasma concentration of Cr, BUN, and SDMA.

Finally, the optimal cutoff value for the K/AO ratio was 10.71, with sensitivity and specificity for identifying 80% and 80% CKD cats, respectively (K/AO ratio ≤10.71). The K/L2 ratio cutoff value was 2.27, with 67% sensitivity and 73% specificity for identifying CKD cats (K/L2 ratio ≤2.27). The K/AO cutoff had higher sensitivity, specificity, and correlation with renal function than the K/L2 ratio.

### Limitations of the study

A major limitation of this study was the small sample size of CKD cats. Further studies, including a large number of cats with early and late IRIS should be conducted to provide further information. In addition, a prospective and multicentric study with a large amount of varied population could be more clarify the usefulness of this preliminary information.

## Conclusion

Our preliminary study showed that the K/AO ratio could be a better parameter for kidney size evaluation in feline CKD than the K/L2 ratio. This parameter was not affected by age, BW, and gender. Therefore, the K/AO ratio could be a practical and promising parameter for assessing kidneys in cats with CKD.

## Authors’ Contributions

NC, KS, DD, NS, and CT: Study conception and design. KJ and CT: Acquisition of data. KJ, NC, and CT: Analysis and interpretation of data and drafting of the manuscript. KJ, NC, KS, DD, NS, and CT: Critical revision. All authors have read, reviewed, and approved the final manuscript.
